# Altered Mitochondrial Function and Oxidative Stress in Leukocytes of Anorexia Nervosa Patients

**DOI:** 10.1371/journal.pone.0106463

**Published:** 2014-09-25

**Authors:** Victor M. Victor, Susana Rovira-Llopis, Vanessa Saiz-Alarcon, Maria C. Sangüesa, Luis Rojo-Bofill, Celia Bañuls, Rosa Falcón, Raquel Castelló, Luis Rojo, Milagros Rocha, Antonio Hernández-Mijares

**Affiliations:** 1 Foundation for the Promotion of Healthcare and Biomedical Research in the Valencian Community (FISABIO), Valencia, Spain; 2 Service of Endocrinology, University Hospital Doctor Peset, Valencia, Spain; 3 Institute of Health Research INCLIVA, University of Valencia, Valencia, Spain; 4 Psychiatry Service, University Hospital La Fe, Department of Medicine, University of Valencia, Valencia, Spain; 5 Research Group CIBER CB/06/02/0045, CIBER actions, Epidemiology and Public Health, University of Valencia, Valencia, Spain; 6 Department of Medicine, University of Valencia, Valencia, Spain; University of Windsor, Canada

## Abstract

**Context:**

Anorexia nervosa is a common illness among adolescents and is characterised by oxidative stress.

**Objective:**

The effects of anorexia on mitochondrial function and redox state in leukocytes from anorexic subjects were evaluated.

**Design and setting:**

A multi-centre, cross-sectional case-control study was performed.

**Patients:**

Our study population consisted of 20 anorexic patients and 20 age-matched controls, all of which were Caucasian women.

**Main outcome measures:**

Anthropometric and metabolic parameters were evaluated in the study population. To assess whether anorexia nervosa affects mitochondrial function and redox state in leukocytes of anorexic patients, we measured mitochondrial oxygen consumption, membrane potential, reactive oxygen species production, glutathione levels, mitochondrial mass, and complex I and III activity in polymorphonuclear cells.

**Results:**

Mitochondrial function was impaired in the leukocytes of the anorexic patients. This was evident in a decrease in mitochondrial O_2_ consumption (P<0.05), mitochondrial membrane potential (P<0.01) and GSH levels (P<0.05), and an increase in ROS production (P<0.05) with respect to control subjects. Furthermore, a reduction of mitochondrial mass was detected in leukocytes of the anorexic patients (P<0.05), while the activity of mitochondrial complex I (P<0.001), but not that of complex III, was found to be inhibited in the same population.

**Conclusions:**

Oxidative stress is produced in the leukocytes of anorexic patients and is closely related to mitochondrial dysfunction. Our results lead us to propose that the oxidative stress that occurs in anorexia takes place at mitochondrial complex I. Future research concerning mitochondrial dysfunction and oxidative stress should aim to determine the physiological mechanism involved in this effect and the physiological impact of anorexia.

## Introduction

Eating disorders are an increasingly frequent pathology that usually affects young women [1] and whose risk factors are varied and complex [2], [3]. Three clinical subtypes of anorexia have been recognised: anorexia nervosa, bulimia nervosa and eating disorders not otherwise classified (EDNOS), which include atypical or incomplete forms of the first two subtypes [4].

Anorexia nervosa (AN), the most severe of the three subtypes, is characterised by a significant and deliberate loss of weight, a distorted perception of one’s body and a pathological fear of being fat [4]. It is often a chronic condition, especially in patients that have required hospital treatment [5], and is currently one of the most frequent disorders among adolescents [6]. Mortality is high [7], and associated physical complications are common, particularly those of a cardiovascular nature [8].

Peripheral polymorphonuclear leukocytes (PMN) are inflammatory cells that, once activated, can release molecules that contribute to inflammation, endothelial impairment and oxidative stress. They also generate excessive amounts of ROS, which are harmful to cells, as they can initiate lipid peroxidation and apoptosis [9]. These effects of ROS are neutralised by the complex antioxidant system developed by organisms. In this context, it has been reported that PMN are contributors to the underlying oxidative stress present in inflammatory diseases and related to mitochondrial dysfunction [10]–[11]. However, their function and redox state in AN patients have not yet been determined.

Mitochondria are an important source of the ROS generated by different complexes, particularly complexes I and III [12]–[13]. For example, in a study performed using a mouse model of anorexia (anx/anx strain), different symptoms were related with mitochondrial impairment in complex I, including poor feeding, neurodegeneration and muscle weakness [14]–[15].

The present study highlights an impairment of mitochondrial function in a population of anorexic patients. This impairment was evident in a decrease in mitochondrial O_2_ consumption, mitochondrial membrane potential (ΔΨ_m_) and glutathione (GSH) levels, and an increase in ROS production. In addition, we observed a reduction of leukocyte mitochondrial mass and an impairment of mitochondrial complex I activity in this patient population.

## Materials and Methods

### Study design

The present multi-centre, cross-sectional case-control study was performed exclusively in Caucasian women. First, the patient/volunteer completed a questionnaire about her menstruation, eating habits, self-perception, influence on life of eating behaviour, binges, regulation of body weight and purging behaviour, and medication. Subsequently, anthropometrical measurements and blood pressure were recorded. A fasting blood sample was taken from all subjects.

### Subjects

Twenty female AN patients with an age range of 16 to 34 (21.2±5.9) years were recruited at the Eating Disorders Unit of the La Fe University Hospital, Valencia. Patients were diagnosed according to the F 50.0 Anorexia nervosa criteria [307.1] of the Diagnostic and Statistical Manual of Mental Disorders (version DSM IV TR): i.e. BMI <18 Kg/m^2^. The presence of known somatic causes of malnutrition and other diseases that could have had a bearing on a subject’s physical condition were ruled out by consulting the patient’s medical history.

The control group consisted of twenty healthy women with an age range of 17 to 33 (23.6±2.8) years, and which were pair-matched with the patients according to age. Controls were recruited at the Outpatient’s Department of the Endocrinology Service of the Dr. Peset University Hospital in Valencia. Eating disorders, recent alterations in body weight, obesity and other metabolic disturbances that could interfere with the study’s objectives were ruled out.

Exclusion criteria were pregnancy or lactation, galactorrhea or any endocrine or systemic disease that could affect reproductive physiology, organic, malignant, haematological, infectious or inflammatory disease, diabetes mellitus, a history of cardiovascular disease and the taking of lipid-lowering or antihypertensive drugs. None of the controls was taking antioxidant supplements at the time. The study was conducted according to the guidelines laid down in the Declaration of Helsinki, and all procedures were approved by the Ethics Committee of the University Hospital Dr Peset. Written informed consent was obtained from all the participants.

A parent or guardian provided written informed consent on behalf of children/minors included in the study.

### Body variables and anthropometrical methods

The following anthropometrical parameters were recorded as specified: height was evaluated with a stadiometer with a variation of 0.4 cm; weight was measured using electronic scales with a variation of 0.05 kg and a capacity of up to 220 kg; BMI was evaluated by dividing weight in kilograms by height^2^ (m); blood pressure was evaluated by using a sphygmomanometer. Waist and hip circumferences were also measured.

### Laboratory methods

Blood was collected from the antecubital vein at 8–10 a.m, following 12 hours of fasting. Glucose levels were measured using enzymatic techniques and a Dax-72 autoanalyzer (Bayer Diagnostic, Tarrytown, New York, USA). Insulin was measured by an enzymatic luminescence technique. Samples for insulin were processed immediately and frozen until analysis in order to avoid haemolysis. Insulin resistance was calculated according to homeostasis model assessment (HOMA) using baseline glucose and insulin: HOMA = (fasting insulin (µU/ml)×fasting glucose (mmol/L)/22.5.

Total cholesterol and triglycerides were measured by means of enzymatic assays, and HDLc concentrations were recorded using a direct method with a Beckman LX-20 autoanalyzer (Beckman Coulter, La Brea, CA, USA). The intraserial variation coefficient was <3.5% for all determinations. LDLc concentration was calculated using the Friedewald method. Non-HDLc concentration was determined based on the difference between total cholesterol and HDLc. Apolipoprotein AI (Apo AI) and B (Apo B) were calculated by immunonephelometry (Dade Behring BNII, Marburg, Germany) with an intra-assay variation coefficient of <5.5%.

### Cells

Human polymorphonuclear leukocytes (PMNs) were obtained from blood samples treated with citrate and incubated with dextran (3%, 45 min). The supernatant was released over Fycoll-Hypaque and centrifuged for 25 min at 250 g. The pellet was resuspended in lysis buffer and centrifuged at room temperature (100 g, 5 min), and was then washed and resuspended in Hank's Balanced Salt Solution (HBSS). PMNs were then counted in a Scepter 2.0 cell counter (Millipore, MA, USA).

### Measurement of O_2_ consumption, membrane potential (ΔΨm) and mitochondrial mass

PMNs were resuspended (5×10^6^ cells/mL) in HBSS medium and placed in a gas-tight chamber. A Clark-type O_2_ electrode (Rank Brothers, Bottisham, UK) was employed to evaluate mitochondrial O_2_ consumption [16]. Sodium cyanide (10^–3^ mol/l), a mitochondrial complex IV inhibitor, was used as a negative control. Measurements were recorded using the Duo.18 data-device (WPI, Stevenage, UK). Rate of O_2_ consumption (V_O2max_) was calculated with the Graph Pad programme. The fluorescent dye tetramethylrhodamine methyl ester (TMRM, 5×10^−6 ^mol/L) was used to assess ΔΨ_m._ Mitochondrial mass was measured using the fluorescent dye 10-N-nonyl acridine orange (NAO, 5×10^−6 ^mol/L), which binds to cardiolipin independently of ΔΨ_m_ [17]. When evaluated by means of the trypan blue exclusion test and Scepter 2.0 cell counter (Millipore, MA, USA), cell viability was found to be unaltered.

### Measurement of ROS production and GSH content

Total ROS production was evaluated by fluorometry using a Synergy Mx plate reader (BioTek Instruments, Winooski, VT) following incubation (30 min) with 5×10^−6^ mol/l of the fluorescent probe 2′,7′-dichlorodihydrofluorescein diacetate (DCFH-DA, 5×10^−6 ^mol/L; excitation 485/emission 535 nm), as described elsewhere [16]. GSH content was calculated following incubation (30 min) with the fluorochrome 5-Chloromethylfluorescein Diacetate (CMFDA, 2.5×10^−6 ^mol/L; excitation 492/emission 517 nm). In short, cells were seeded on 96-well plates, washed with phosphate-buffered saline and incubated with CMFDA diluted in phosphate-buffered saline. After 15 min at 37°C, fluorescence intensities were measured. Levels of ROS and intracellular GSH were expressed as arbitrary fluorescence units.

### Mitochondrial Respiratory Chain Enzyme Activities

Cell pellets containing approximately 10×10^6^ cells were harvested, resuspended in 0.5 ml of Buffer A (20 mM MOPS, 0.25 M sucrose), centrifuged at 5000 g for 3 minutes at 4°C, resuspended in Buffer B (20 mM MOPS, 0.25 M sucrose, 1 mM EDTA), centrifuged at 10000 g for 3 minutes at 4°C and resuspended in 200 µL of 10 mM KH_2_PO_4_ (pH 7.4). Protein extracts where sonicated for 10 seconds in an Ultrasons cleaner (JP Selecta S.A., Barcelona, Spain). The protein concentration of each sample was determined by the BCA method, as described by the supplier (Pierce, Rockford, IL).

NADH oxidation was evaluated in a cuvette at 340 nm in a dual beam U-2800 spectrophotometer at 30°C. 35 µg of sample were added to 1000 µL of reaction buffer containing 20 mM KH_2_PO_4 _pH 8, 200 µM NADH, 1 mM NaN_3_ and 0.1% BSA. First, a baseline rate was recorded for 2 min. in the absence of the substrate. Ubiquinone was then added to a final concentration of 100 µM and the rate of NADH oxidation was recorded for 3 min. NADH oxidation is produced by mitochondrial Complex I and other cellular NADH dehydrogenases. In order to determine Complex I activity, rotenone, an inhibitor of Complex I, was added to a final concentration of 5 µM and the rate of NADH oxidation was determined for another 3 min. Complex I activity was calculated by subtracting the rotenone-insensitive NADH oxidation rate in its linear phase from the total NADH oxidation rate in its linear phase. Complex III activity was measured by cytochrome C reduction at 550 nm at 30°C. 20 µg of protein were added to 1000 µL of reaction buffer containing 50 mM KH_2_PO_4 _pH 7.5, 2 mM NaN_3_, 0.1%, BSA, 50 µM cytochrome C and 50 µM decylubiquinone, with and without 10 µg/ml antimycin A. Complex III activity was calculated by subtracting the antimycin A-insensitive cytochrome C reduction rate from the total cytochrome C reduction rate in the absence of antimycin A. The activity of the mitochondrial respiratory chain complexes was expressed as nmol min^−1 ^mg protein^−1^ and converted to a % of that of controls. Residual activity of the different respiratory chain complexes in the presence of specific inhibitors was always less than 10% that of controls. Human U937 leukocytic cells treated previously with rotenone (10 µM for 12 h) or antimycin A (10 µM for 2 h) were used as controls for complex I and complex III inhibition, respectively.

### Drugs and solutions

Sodium cyanide, trypan blue, MOPS, BSA, Coenzyme Q1, rotenone, sodium azide (NaN_3_), cytochrome C, antimycin A, decylubiquinone and HBSS were purchased from Sigma-Aldrich (Sigma Chem. Co., St. Louis, MO, USA). NADH was purchased from Roche Applied Science (Mannheim, Germany). Dextran was provided by Fluka (St. Louis, MO, USA). DCFH-DA was provided by Calbiochem (San Diego, CA, USA). PBS, TMRM, CMFDA and NAO were supplied by Invitrogen (Eugene, OR, USA). Ficoll-Paque TM Plus was purchased from GE Healthcare (Little Chalfont, Buckinghamshire, UK).

### Statistical analyses

Statistical analysis was performed with SPSS 17.0 software (SPSS Statistics Inc., Chicago, IL, USA). Continuous variables were expressed as mean and standard deviation (SD) or as median or 25th and 75th percentiles for parametric and non-parametric data, respectively. Patient and control data were compared using a Student’s t test for parametric independent samples or a Mann-Whitney U-test for non-parametric samples. Pearson's correlation or Spearman’s correlation coefficients was employed to measure the strength of the association between two variables for parametric and non-parametric data, respectively. All the tests used a confidence interval of 95% and differences were considered significant when P<0.05.

## Results

### Anthropometric, metabolic and clinical characteristics

The anthropometric characteristics of control subjects and anorexic patients can be seen in Table 1, which shows lower (P<0.001) weight, BMI and waist circumference among the latter. Systolic blood pressure was also lower (P<0.05) in anorexic patients.

**Table 1:** Baseline characteristics and anthropometric data for the women participating in the study.

	Controls (n = 20)	Anorexia nervosa (n = 20)	p-value
Age (years)	23.6±2.8	21.2±5.9	0.210
Weight (kg)	56.8±3.6	40.3±4.1	<0.001
BMI (kg/m^2^)	20.6±0.6	15.3±0.9	<0.001
Waist circumference (cm)	72.0±4.3	63.4±3.6	<0.001
Systolic blood pressure (mmHg)	107±8	97±9	0.010
Diastolic blood pressure (mmHg)	66±7	65±7	0.833

Comparison between anorexic patients and controls using an unpaired Student’s t-test. Data are expressed as mean ± SD. n = 20.

Endocrine parameters in anorexic patients and their respective controls are shown in Table 2. Decreases in insulin levels (P<0.001) and HOMA-IR (P<0.001) were observed in the anorexic group. When assessing potential correlations between BMI and lipid parameters in the anorexic women, it was found that BMI and weight were not associated with any of the parameters analysed (CT, LDLc, HDLc and triglycerides). In contrast, BMI correlated with total cholesterol (r = −0.295; p = 0.027) and HDLc (r = −0.323, p = 0.015) when the whole population was considered (controls and patients).

**Table 2:** Endocrine parameters in anorexia nervosa (AN) patients and healthy control subjects.

	Controls	Anorexia nervosa	p-value
Total cholesterol (mg/dl)	178.6±30.1	192.1±43.3	0.378
LDLc (mg/dl)	105.5±21.5	106.9±37.6	0.910
HDLc (mg/dl)	61.7±10.7	71.2±14.8	0.079
Triglycerides (mg/dl)	56 (46,61.5)	71 (38.5,87.5)	0.368
Apolipoprotein AI (mg/dl)	158.5±25.6	140.1±29.2	0.117
Apolipoprotein B (mg/dl)	74.8±12.8	73.5±26.3	0.884
Glucose (mg/dl)	80.4±8.9	75.0±6.0	0.083
Insulin (µIU/ml)	6.50±1.98	2.54±1.66	<0.001
HOMA-IR	1.29±3.77	0.49±0.39	<0.001

Data are expressed as mean ± SD, except for triglycerides, which are represented as medians and IQ range. Values of serum triglyceride concentrations were normalized using a log transformation. Comparison between anorexic patients and controls using an unpaired Student’s t-test. n = 20.

### Mitochondrial O_2_ consumption, ΔΨm and mitochondrial mass

**Figure 1:**
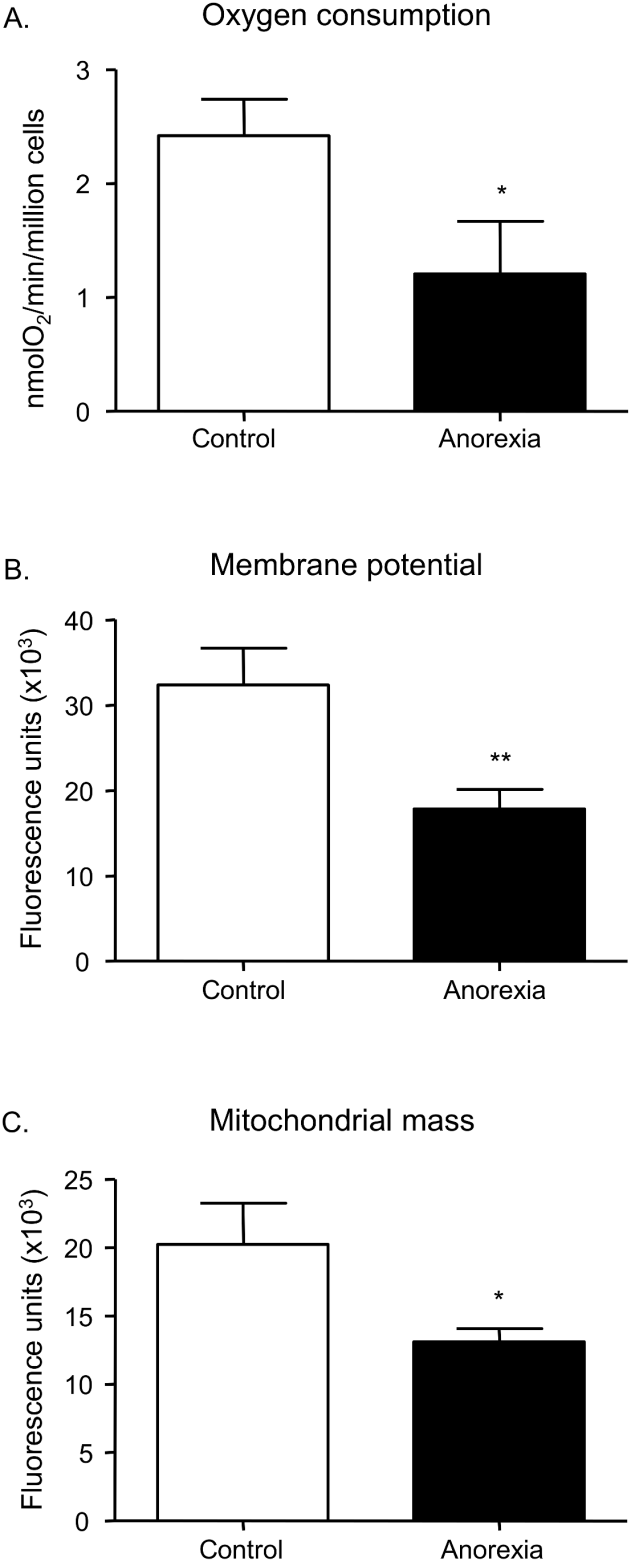
Effect of anorexia on O_2_ consumption (A) rate, measured as nmol O_2_/min/million cells, on membrane potential (B), measured by TMRM fluorescence, and on mitochondrial mass (C), measured by NAO fluorescence. n = 20 per group. *P<0.05 and **P<0.01 *vs.* control.

A Clark electrode with an O_2_-tight chamber was used to evaluate the rate of mitochondrial O_2_ consumption in PMNs from the blood of both anorexic patients and controls. O_2_ consumption proved to be mitochondrial, since the presence of sodium cyanide inhibited O_2_ consumption (95–99%; not shown). The rate of O_2_ consumption was lower in anorexic patients than in controls (Fig. 1A, P<0.05). Both TMRM (P<0.01) and NAO (P<0.05) fluorescence were diminished in anorexic patients (P<0.05), the former indicating a reduction in Δψ*_m_* (Fig. 1B) and the latter a reduction in mitochondrial mass (Fig. 1C).

### ROS production and GSH content

**Figure 2:**
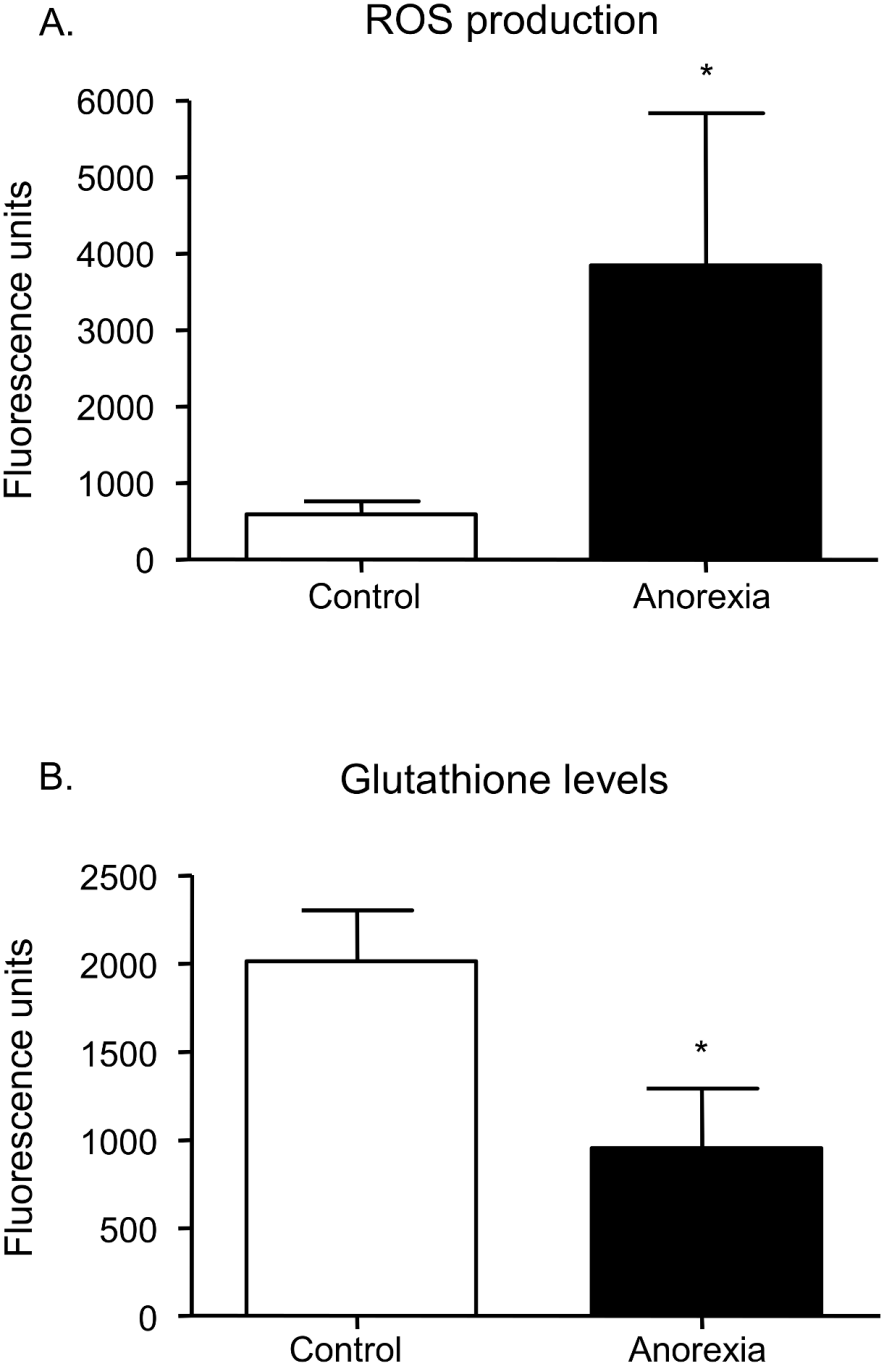
Effect of anorexia on ROS production (A), measured by DCFH fluorescence, and on GSH content (B), measured by CMFDA fluorescence. n = 20 per group. *P<0.05 *vs.* controls.

DCFH-DA fluorescence was significantly higher (Fig. 2A, P<0.05) and levels of GSH were significantly lower (Fig. 2B, P<0.05) among anorexic patients. These results confirmed an oxidative stress pattern in this group.

### Mitochondrial complex I and III activity

**Figure 3:**
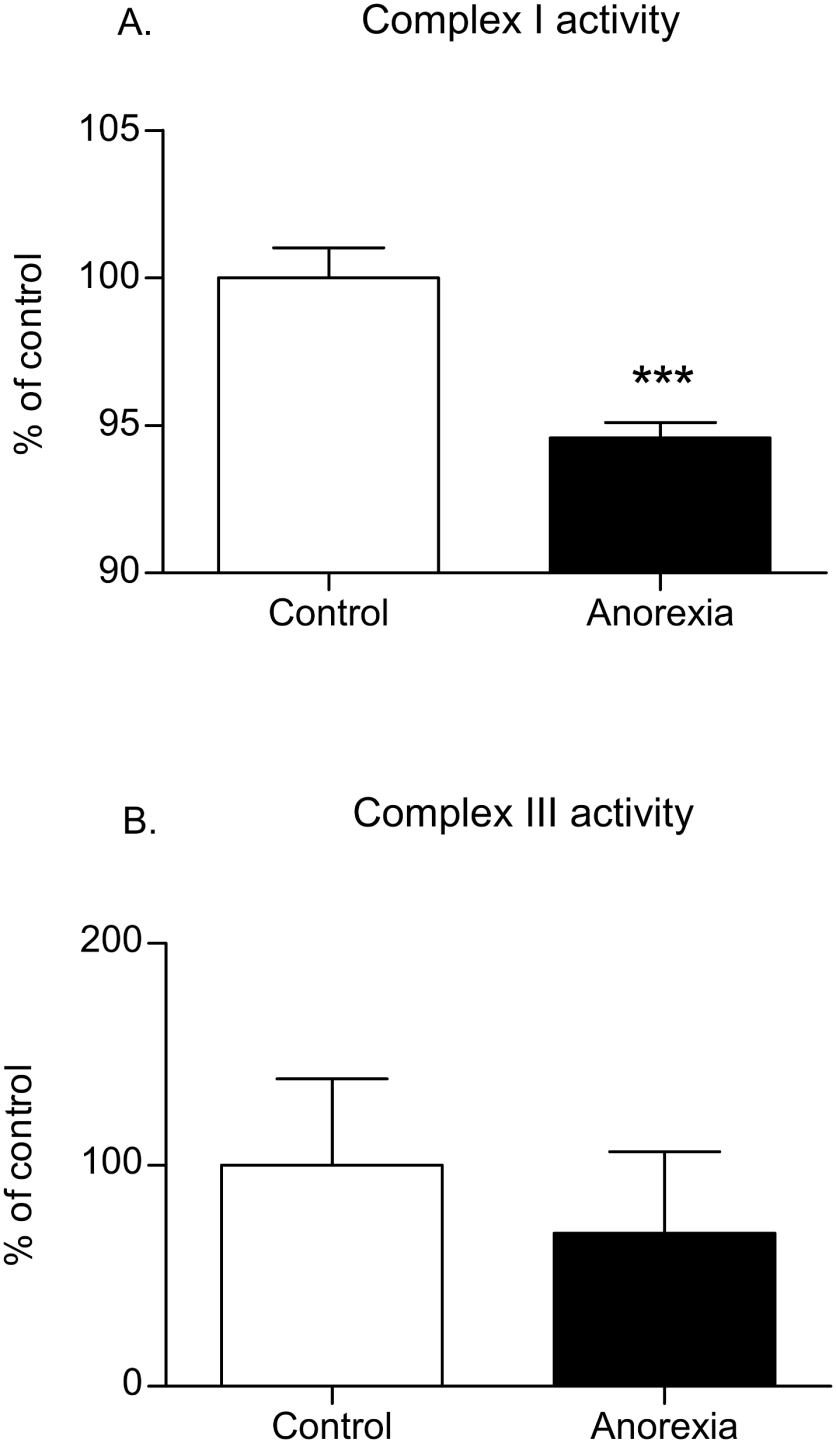
Effect of anorexia on mitochondrial complex I activity (A), measured as the NADH oxidation rate at 340 nm, and on complex III activity (B), measured as the cytochrome C reduction rate at 550 nm. Specific inhibitors for complexes I and III, rotenone and antimycin A, respectively, were employed to ensure the specificity of the assay. n = 8 per group. ***P<0.001 *vs.* control.

As mitochondrial complexes I and III continuously generate ROS, they are particularly susceptible to oxidative damage. Therefore, we assessed the activity of both complexes in order to elucidate whether they were altered or not. Figure 3A shows the inhibition of mitochondrial complex I activity in anorexic patients (P<0.001), calculated as the rate of NADH oxidation in PMNs (expressed as % of control activity). The data suggest that complex I was the main target of the mitochondrial dysfunction in PMNs from the patients. No differences in complex III activity were detected between the anorexic and control groups (Fig. 3B). Human U937 leukocytes treated with rotenone or antimycin A were employed as controls and confirmed the inhibition of complex I and complex III respectively (P<0.001, data not shown).

## Discussion

The present study highlights an impairment of mitochondrial function in PMNs from anorexic patients, expressed as a decrease in mitochondria O_2_ consumption, ΔΨ_m_ and GSH levels, and an increase in ROS production. We also demonstrate a reduction in mitochondrial mass in leukocytes from the same patients. Additionally, we propose complex I of the electron transport chain as a target of the ROS which are generated during anorexia. In line with this, we have previously demonstrated that complex I is more susceptible to oxidation than other mitochondrial complexes under oxidative stress conditions [16].

The mitochondrial electron transport chain is the principal site of ATP and ROS production in cells. In mammalian cells under normal conditions, mitochondrial complex I (CI) and mitochondrial complex III (CIII) are the main intracellular sources of the ROS generated by leaking electrons. In addition, ROS play several roles in cell and metabolic signalling [18]–[19]. Levels of ROS can increase dramatically during certain conditions, such as complex I dysfunction [10], [16], [20]–[21], resulting in oxidative stress and apoptosis [22]. In this context, an increase of ROS has previously been described in an animal model of anorexia – anx/anx mice [15] - which is in line with our observations of a decrease in O_2_ consumption and ΔΨ_m_ and an increase of ROS in human anorexic subjects. High ROS production has been related to the pathology of a high number of diseases, including atherosclerosis, cardiovascular disease, cancer and diabetes [13], [15], [17], [23]–[24]. However, in addition to their status as oxidative molecules, ROS also act as metabolic signalling molecules [25]. The authors of the aforementioned study performed with the anx/anx mouse model hypothesized that a subclinical mitochondrial complex I deficiency increases ROS production and damages sensitive hypothalamic neurons, thereby inducing an impairment of appetite-regulating neuronal networks.

The present study highlights an increase in ROS production in the leukocytes of anorexic patients, which is relevant given that leukocytes are known to be highly sensitive to oxidative damage [26]–[27]. In fact, excessive ROS levels can be deleterious to cells, as they induce lipid peroxidation and apoptosis. However, these harmful effects of ROS can be counteracted by the organism’s antioxidant system; antioxidants in general, and particularly GSH, play a key role in maintaining the viability of cells and protecting them from high levels of ROS/oxidative stress [28]–[30]. Malnutrition is usually accompanied by deficiency of vitamins and antioxidants; in fact, an undermined antioxidant status has been reported in AN [31], which supports the data of the present study. Furthermore, previous studies have shown that oxidative stress can accelerate hepatocyte injuries provoked during anorexia by increasing lipid peroxidation [32].

The increase in ROS production and decrease in GSH levels, mitochondrial O_2_ consumption and ΔΨ_m_ in our anorexic subjects point to an impairment of the electron transport chain that alters the function of mitochondria as a source of ATP. Moreover, we have witnessed a decrease of mitochondrial mass in these patients, which is relevant, as mitochondrial mass and morphology are important mediators of mitochondrial function [33]–[34]. A reduction in mitochondrial mass has been observed in different diseases, including diabetes [35], [17], and hyperglycemia has been shown to induce mitochondrial fission, high ROS production and a reduction of ATP [36].

Importantly, while our anorexic patients exhibited lower mitochondrial mass than controls, they displayed a higher ROS production, indicating a pro-oxidant state despite the reduced amount of mitochondria. ROS production can disturb the GSH/GSSG ratio and redox state by reacting with thiol residues within redox-sensitive proteins. Our data show that AN leads to a decrease in GSH levels in leukocytes. In addition, we demonstrate that activity of complex I, but not of complex III, is impaired in anorexic patients, possibly due to high ROS production. In relation to this, different symptoms of anorexia have recently been linked to mitochondrial impairment in complex I in the previously mentioned study in a mouse model of anorexia (anx/anx mice), including poor feeding, neurodegeneration and muscle weakness [14]–[15].

Considered together, these findings confirm a state of oxidative stress in anorexic patients. PMNs are extremely sensitive under oxidative stress conditions in several diseases, including type 2 diabetes [10] and polycystic ovary syndrome [16]. In addition, oxidative stress can activate numerous inflammatory mediators, such as adhesion molecules, and proinflammatory cytokine expression (e.g. TNF-α, whose levels are enhanced in anorexia) [37] and immune signaling responses, including phospholipase activity, MAP kinase, and STAT and TLR signaling pathways [38]. In fact, TNF-α has been shown to mediate weight loss in experimental animal models through several mechanisms, including catabolic effects on energy storage tissue, suppression of food intake and lipoprotein lipase inhibition [37]. In light of this evidence, PMNs would seem to be one of the main types of inflammatory cells. Once activated, they release proinflammatory cytokines and ROS, which contribute to mitochondrial and endothelial dysfunction, reticulum stress, oxidative stress, inflammation and cardiovascular diseases (CVD) [10], [16].

In summary, the present study demonstrates that oxidative stress is produced in the leukocytes of anorexic patients and that this stress is closely related to mitochondrial dysfunction. In fact, we show that mitochondrial complex I is inhibited in anorexic patients. Future research concerning mitochondrial dysfunction and oxidative stress should aim to determine the physiological mechanism involved in this effect and the physiological impact of anorexia.

## References

[1] Rojo L, Livianos L, Conesa L, García A, Domínguez A, et al (2003) Epidemiology and risk factors of eating disorders. A two stage epidemiological study in a spanish population aged 12–18 years. Int J Eat Disord 34: 281–291. 12949920 10.1002/eat.10179

[2] Fairburn CG, Harrison PJ (2003) Eating disorders. Lancet 361: 407–416. 12573387 10.1016/S0140-6736(03)12378-1

[3] Ghaderi A (2003) Structural modeling analysis of prospective risk factors for eating disorder. Eating Behaviors 3: 387–396. 15000998 10.1016/s1471-0153(02)00089-2

[4] American Psychiatric Association (1994) Diagnostic and statistical manual of mental disorders. 4^th^ ed. Washington DC: American Psychiatric Press.

[5] Keel PK, Brown TA (2010) Update on Course and Outcome in Eating Disorders. Int J Eat Disord 43: 195–204. 20186717 10.1002/eat.20810

[6] Fisher M (2006) Treatment of eating disorders in children, adolescents, and young adults. Pediatr Rev 27: 5–16. 16387924 10.1542/pir.27-1-5

[7] Neumärker KJ (1997) Mortality and sudden death in anorexia nervosa. Int J Eat Disord 21: 205–212. 9097194 10.1002/(sici)1098-108x(199704)21:3<205::aid-eat1>3.0.co;2-o

[8] Katzman DK (2005) Medical complications in adolescent with anorexia nervosa: a review of the literature. Int J Eat Disord 37: 552–559. 10.1002/eat.20118 15852321

[9] Victor VM, Rocha M, De la Fuente M (2004) Immune cells: free radicals and antioxidants in sepsis. Int Immunopharmacol 4: 327–347. 15037211 10.1016/j.intimp.2004.01.020

[10] Hernandez-Mijares A, Rocha M, Apostolova N, Borras C, Jover A, et al (2011) Mitochondrial complex I impairment in leukocytes from type 2 diabetic patients. Free Radic Biol Med 50: 1215–1221. 21262346 10.1016/j.freeradbiomed.2011.01.019

[11] Murphy MP (2009) How mitochondria produce reactive oxygen species. Biochem J 417: 1–13. 19061483 10.1042/BJ20081386 PMC2605959

[12] James AM, Collins Y, Logan A, Murphy MP (2012) Mitochondrial oxidative stress and the metabolic syndrome. Trends Endocrinol Metab 23: 429–434. 22831852 10.1016/j.tem.2012.06.008

[13] Rovira-Llopis S, Rocha M, Falcon R, de Pablo C, Alvarez A, et al (2013) Is Myeloperoxidase a Key Component in the ROS-Induced Vascular Damage Related to Nephropathy in Type 2 Diabetes? Antioxid Redox Signal 19: 1452–1458. 23521574 10.1089/ars.2013.5307 PMC3797450

[14] Luft R, Landau BR (1995) Mitochondrial medicine. J Intern Med 238: 405–421. 7595180 10.1111/j.1365-2796.1995.tb01218.x

[15] Lindfors C, Nilsson I, Garcia-Roves PM, Zuberi AR, Karimi M, et al (2011) Hypothalamic mitochondrial dysfunction associated with anorexia in the anx/anx mouse. PNAS 108: 18108–18113. 22025706 10.1073/pnas.1114863108 PMC3207677

[16] Victor VM, Rocha M, Bañuls C, Sanchez-Serrano M, Sola E, et al (2009) Mitochondrial complex I impairment in leukocytes from polycystic ovary syndrome patients with insulin resistance. J Clin Endocrinol Metab 94: 3505–3512. 19567514 10.1210/jc.2009-0466

[17] Hernandez-Mijares A, Rocha M, Rovira-Llopis S, Bañuls C, Bellod L, et al (2013) Human leukocyte/endothelial cell interactions and mitochondrial dysfunction in type 2 diabetic patients and their association with silent myocardial ischemia. Diabetes Care 36: 1695–1702. 23300290 10.2337/dc12-1224 PMC3661843

[18] Schagger H, de Coo R, Bauer MF, Hofmann S, Godinot C, et al (2004) Significance of respirasomes for the assembly (stability of human respiratory complex I. J Biol Chem 2004. 279: 36349–36353. 10.1074/jbc.M404033200 15208329

[19] Grad LI, Lemire BD (2006) Riboflavin enhances the assembly of mitochondrial cytochrome c oxidase in C elegans NADH-ubiquinone oxidoreductase mutants. Biochim Biophys Acta 1757: 115–122. 16443191 10.1016/j.bbabio.2005.11.009

[20] Ugalde C, Janssen RJ, van den Heuvel LP, Smeitink JA, Nijtmans LG (2004) Differences in assembly or stability of complex I and other mitochondrial OXPHOS complexes in inherited complex I deficiency. Hum Mol Genet 13: 659–667. 14749350 10.1093/hmg/ddh071

[21] Victor VM, Rocha M, Bañuls C, Alvarez A, de Pablo C, et al (2011) Induction of oxidative stress and human leukocyte/endothelial cell interactions in polycystic ovary syndrome patients with insulin resistance. J Clin Endocrinol Metab 96: 3115–3122. 21778215 10.1210/jc.2011-0651

[22] Barrientos A, Moraes CT (1999) Titrating the effects of mitochondrial complex I impairment in the cell physiology. J Biol Chem 274: 16188–16197. 10347173 10.1074/jbc.274.23.16188

[23] Esposito LA, Melov S, Panov A, Cottrell BA, Wallace DC (1999) Mitochondrial disease in mouse results in increased oxidative stress. Proc Natl Acad Sci U S A 96: 4820–4825. 10220377 10.1073/pnas.96.9.4820 PMC21775

[24] Rocha M, Apostolova N, Herance JR, Rovira-Llopis S, Hernandez-Mijares A, et al (2014) Perspectives and potential applications of mitochondria-targeted antioxidants in cardiometabolic diseases and type 2 diabetes. Med Res Rev 34: 160–189. 23650093 10.1002/med.21285

[25] Diano S, Liu ZW, Jeong JK, Dietrich MO, Ruan HB, et al (2011) Peroxisome proliferation-associated control of reactive oxygen species sets melanocortin tone and feeding in diet-induced obesity. Nat Med 17: 1121–1127. 21873987 10.1038/nm.2421 PMC3388795

[26] De la Fuente M, Victor VM (2000) Antioxidants as modulators of immune function. Immunol Cell Biol 78: 49–54. 10651929 10.1046/j.1440-1711.2000.00884.x

[27] Sela S, Mazor R, Amsalam M, Yagil C, Yagil Y, et al (2004) Primed polymorphonuclear leukocytes, oxidative stress, and inflammation antecede hypertension in the Sabra rat. Hypertension 44: 764–769. 15452031 10.1161/01.HYP.0000144480.10207.34

[28] Victor VM, Rocha M, Esplugues JV, De la Fuente M (2005) Role of free radicals in sepsis: antioxidant therapy. Curr Pharm Des 11: 3141–3158. 16178750 10.2174/1381612054864894

[29] Yang YT, Whiteman M, Gieseg SP (2012) Intracellular glutathione protects human monocyte-derived macrophages from hypochlorite damage. Life Sci 90: 682–688. 22472425 10.1016/j.lfs.2012.03.002

[30] Monsalve M, Borniquel S, Valle I, Lamas S (2007) Mitochondrial dysfunction in human pathologies. Front Biosci 12: 1131–1153. 17127367 10.2741/2132

[31] Moyano D, Sierra C, Brandi N, Artuch R, Mira A, et al (1999) Antioxidant status in anorexia nervosa. Int J Eat Disord 25: 99–103. 9924658 10.1002/(sici)1098-108x(199901)25:1<99::aid-eat12>3.0.co;2-n

[32] Tajiri K, Shimizu Y, Tsuneyama K, Sugiyama T (2006) A case report of oxidative stress in a patient with anorexia nervosa. Int J Eat Disord 39: 616–618. 16927384 10.1002/eat.20326

[33] Yoon Y (2005) Regulation of mitochondrial dynamics: another process modulated by Ca^2+^ signals? Sci STKE 2005: pe18. 15840838 10.1126/stke.2802005pe18

[34] Riva A, Tandler B, Loffredo F, Vazquez E, Hoppel C (2005) Structural differences in two biochemically defined populations of cardiac mitochondria. Am J Physiol Heart Circ Physiol 289: H868–H872. 15821034 10.1152/ajpheart.00866.2004

[35] Morino K, Petersen K, Dufour S (2005) Reduced mitochondrial density and increased IRS-1 serine phosphorylation in muscle of insulin-resistant offspring of type 2 diabetic parents. J Clin Invest 115: 3587–3593. 16284649 10.1172/JCI25151 PMC1280967

[36] Yu T, Robotham JL, Yoon Y (2006) Increased production of reactive oxygen species in hyperglycemic conditions requires dynamic change of mitochondrial morphology. Proc Natl Acad Sci U S A 103: 2653–2658. 16477035 10.1073/pnas.0511154103 PMC1413838

[37] Tracey KJ, Morgello S, Koplin B, Fahey TJ 3rd, Fox J, et al (1990) Metabolic effects of cachectin/tumor necrosis factor are modified by site of production. Cachectin/tumor necrosis factor-secreting tumor in skeletal muscle induces chronic cachexia, while implantation in brain induces predominantly acute anorexia. J Clin Invest 86: 2014–24. 2254457 10.1172/JCI114937 PMC329839

[38] Ivison SM1, Wang C, Himmel ME, Sheridan J, Delano J, et al. (2010) Oxidative stress enhances IL-8 and inhibits CCL20 production from intestinal epithelial cells in response to bacterial flagellin. Am J Physiol Gastrointest Liver Physiol 299: G733–741. 20595617 10.1152/ajpgi.00089.2010

